# Biomarker Analysis Revealed Distinct Profiles of Innate and Adaptive Immunity in Infants with Ocular Lesions of Congenital Toxoplasmosis

**DOI:** 10.1155/2014/910621

**Published:** 2014-09-18

**Authors:** Anderson Silva Machado, Ana Carolina Aguiar Vasconcelos Carneiro, Samantha Ribeiro Béla, Gláucia Manzan Queiroz Andrade, Daniel Vitor Vasconcelos-Santos, José Nélio Januário, Jordana G. Coelho-dos-Reis, Eloisa Amália Vieira Ferro, Andréa Teixeira-Carvalho, Ricardo Wagner Almeida Vitor, Olindo Assis Martins-Filho, UFMG Congenital Toxoplasmosis Brazilian Group —UFMG-CTBG

**Affiliations:** ^1^Departamento de Parasitologia, Universidade Federal de Minas Gerais, Avenida Presidente Antônio Carlos, 6627, Pampulha, 31270-901 Belo Horizonte, MG, Brazil; ^2^Laboratório de Biomarcadores de Diagnóstico e Monitoração, Centro de Pesquisas René Rachou, Fundação Oswaldo Cruz, Avenida Augusto de Lima, 1715 Barro Preto, 30190-002 Belo Horizonte, MG, Brazil; ^3^Departamento de Pediatria, Universidade Federal de Minas Gerais, Avenida Professor Alfredo Balena 190, Santa Efigênia, 30130-100 Belo Horizonte, MG, Brazil; ^4^Departamento de Oftalmologia e Otorrinolaringologia, Faculdade de Medicina da UFMG, Belo Horizonte, MG, Brazil; ^5^Núcleo de Ações e Pesquisa em Apoio Diagnóstico (NUPAD), Universidade Federal de Minas Gerais, Avenida Professor Alfredo Balena 190, Santa Efigênia, 30130-100 Belo Horizonte, MG, Brazil; ^6^Universidade Federal de Uberlândia, Avenida João Naves de Ávila 2121, Santa Mônica, 38408-100 Uberlândia, MG, Brazil

## Abstract

*Toxoplasma gondii* is the main infectious cause of human posterior retinochoroiditis, the most frequent clinical manifestation of congenital toxoplasmosis. This investigation was performed after neonatal screening to identify biomarkers of immunity associated with immunopathological features of the disease by flow cytometry. The study included infected infants without NRL and with retinochoroidal lesions (ARL, ACRL, and CRL) as well as noninfected individuals (NI). Our data demonstrated that leukocytosis, with increased monocytes and lymphocytes, was a relevant hematological biomarker of ARL. Immunophenotypic analysis also revealed expansion of CD14^+^CD16^+^HLA-DR^high^ monocytes and CD56^dim^ cytotoxic NK-cells in ARL. Moreover, augmented TCR*γ* *δ* ^+^ and CD8^+^ T-cell counts were apparently good biomarkers of morbidity. Biomarker network analysis revealed that complex and intricated networks underscored the negative correlation of monocytes with NK- and B-cells in NRL. The remarkable lack of connections involving B-cells and a relevant shift of NK-cell connections from B-cells toward T-cells observed in ARL were outstanding. A tightly connected biomarker network was observed in CRL, with relevant connections of NK- and CD8^+^ T-cells with a broad range of cell subsets. Our findings add novel elements to the current knowledge on the innate and adaptive immune responses in congenital toxoplasmosis.

## 1. Introduction

Ocular toxoplasmosis is a common inflammatory eye disease and a major cause of posterior uveitis worldwide [1]. The importance of toxoplasmosis is even greater in Brazil, where the prevalence and severity of ocular disease are higher than those in the rest of the world [2]. A study conducted in Brazil showed that 90% of newborns with congenital toxoplasmosis had clinical signs at birth. The main symptom observed was retinochoroiditis, present in 80% of newborns [3].

The success of infection caused by* T. gondii* is based on a delicate balance between the host immune response, which tries to clear the parasite, and the immune evasion strategies or immunomodulation elicited by the parasite, which enables the ultimate survival of both the parasite and the host [4]. A number of different host cells and compartments are involved in the immune response to* T. gondii,* and the interplay between these cells is crucial to resistance to the parasite [5–9].

Scarce immunological data on ocular disease in humans is available and these studies have mainly focused on the* T. gondii*-specific T-cell response* in vitro*. The analysis of systemic specific cellular response to* T. gondii* antigen in patients without and with active/cicatricial ocular lesions in acquired or congenital disease has described controversial data on the role of proinflammatory response in this scenario. Yamamoto and colleagues have characterized the immune response in adult patients with ocular disease due to congenital infection and have suggested that these patients may show tolerance toward the parasite by decreased proinflammatory response along with lower lymphoproliferative index [10]. In contrast, Fatoohi and colleagues show that systemic cellular response to* T. gondii* does not differ between adult patients without and with presumed congenital ocular toxoplasmosis in regard to T-cell activation and proinflammatory cytokine production [11]. Despite recent advances in toxoplasmosis immunology, relatively little attention has been focused on the immunological events related to the congenital toxoplasmic retinochoroiditis in infant patients. Considering these previous reports, this work aimed at characterizing the* ex vivo* systemic immunophenotypic profile of innate and adaptive cell subsets during the early phases of congenital toxoplasmosis and its association with the absence/presence of active/cicatricial retinochoroiditis in infants.

## 2. Methods

### 2.1. Study Population

The protocols conducted in this study were approved by the local Ethics Committee (Federal University of Minas Gerais, protocol 298/06) and written informed consent was obtained from all mothers of infants included in this study.

This study was part of a prospective investigation on neonatal screening for congenital toxoplasmosis conducted by a multidisciplinary research group (UFMG Congenital Toxoplasmosis Brazilian Group). From November 1, 2006, to May 31, 2007, a total of 146,307 children were tested for anti-*T. gondii* IgM antibodies in dried blood samples on filter paper using the Toxo IgM kit (Q-Preven, Symbiosis, Leme, Brazil). Confirmative plasma/serum tests were run in 220 infants and their mothers in cases with positive or underterminated screening results. The mothers and infants were tested for IgA (enzyme-linked immunosorbent assay) and IgG and IgM anti-*T. gondii* (enzyme-linked fluorometric assay, ELFA-VIDAS, BioMérieux SA, Lyon, France). Out of these 220 cases, 190 infants tested positive by confirmative exams and for the persistence of anti-*T. gondii* IgG antibodies in serum at the age of 12 months. All infants included in this study received medical care by a general clinical physician with experience with infectious diseases and the physical examination did not reveal any alteration. Ophthalmologic evaluation was performed by two retina/uveitis specialists assisted by a trained nursing professional according to a standardized protocol as reported elsewhere [3]. Infants also underwent a through pediatric examination, neuroimaging studies (cranial radiographs or transfontanel ultrasound; computer-assisted tomography in selected cases), hearing assessment, and ophthalmologic evaluation. Peripheral blood samples from 105 infants (45 ± 12 days of age; 53% male, 47% female) were collected to obtain leukocytes. These infants were classified into two groups: (i) group TOXO (infected infants), which comprised 83 infants diagnosed with congenital toxoplasmosis who had positive confirmative tests and persistent specific IgG antibodies, and (ii) group NI (control noninfected infants), which comprised 22 infants who tested negative by IgG anti-*T. gondii*. Among the 83 children from group TOXO, 15 infants presented active retinochoroidal lesions (ARL), 27 had simultaneous active and cicatricial retinochoroidal lesions (ACRL), 17 had cicatricial retinochoroidal lesions (CRL), and 24 had no retinochoroidal lesions (NRL). Infants from the NI group did not have any type of retinochoroidal lesions.

### 2.2. Flow Cytometric Acquisition and Analysis

Peripheral blood from infants with congenital toxoplasmosis (TOXO) and noninfected infants (NI) was processed, and leukocyteswere used for* ex vivo* protocols, as previously described [10]. Monoclonal antibodies (mAbs) were used for labeling cell surface molecules, for T and NKT lymphocytes (anti-CD3, anti-CD4, and anti-CD8), B lymphocytes (anti-CD5, anti-CD19, and anti-CD23), monocytes (anti-CD14, anti-CD16, anti-CD32, and anti-CD64), NK- and NKT-cells (anti-CD16, anti-CD56), anti-HLA-DR (activation), conventional T-cells (anti-TCRα*β*), and gamma-delta T-cells (anti-TCR*γ* *δ*), labeled with fluorescein isothiocyanate (FITC), phycoerythrin (PE), or TRI-COLOR (TC), which were purchased from Invitrogen Life Technologies (Carlsbad, CA, USA).

Cytofluorimetric data acquisition was performed with a Becton Dickinson FACSCalibur instrument. CELLQUEST software provided by the manufacturer was used for data analysis.

### 2.3. Data Analysis

This was a descriptive transversal study that applied three data analysis approaches for observational investigation of the immunological profile associated with distinct clinical manifestations of congenital toxoplasmosis, referred to as (1) conventional statistical analysis, (2) biomarker signature analysis, and (3) biomarkers network. The two later approaches have been shown as relevant to detect, with high sensitivity, putative minor changes in the immunological profiles that are not detectable by conventional statistical approaches.

#### 2.3.1. Conventional Statistical Analysis

Statistical analyses were conducted using GraphPad Prism 5.0 software (GraphPad Software, San Diego, CA, USA). Differences between groups were first evaluated to test the normality. Considering the nonparametric nature of all data sets, statistical analyses between the TOXO and NI groups were performed by the Mann-Whitney test. Additional analyses among the TOXO subgroups were performed by the Kruskal-Wallis test, followed by Dunns' multiple comparison test. Data sets are presented as scatter distributions over median values (bars) for TOXO and NI groups. Data from the TOXO subgroups analysis were presented in box-plot format, highlighting the median together with the minimum and maximum values. In all cases, differences were considered significant at *P* < 0.05.

#### 2.3.2. Biomarker Signature Analysis

The use of this approach was adapted from a pioneering study in order to identify relevant differences in the peripheral blood phenotypic signatures between the groups [11]. In this data analysis, initially, the whole universe of data of each cell subset was used to calculate the global median value used as the cut-off to classify infants as with “low” or “high” counts of a given biomarker. The following cut-offs were used to categorize each infant as presenting “low” or “high” levels of a given cell subset: MONCD16^+^ = 125.0 cells/mm^3^, MONCD16^+^DR^high^ = 86.2 cells/mm^3^, MONCD32^+^ = 78.8 cells/mm^3^, MONCD64^+^ = 186.6 cells/mm^3^, NK-cells = 1195.2 cells/mm^3^, NKCD16^+^CD56^−^ = 441.2 cells/mm^3^, NKCD16^+^CD56^+^ = 526.0 cells/mm^3^, NKCD16^−^CD56^+^ = 85.4 cells/mm^3^, CD3^−^CD56^dim⁡^ = 2.43%, CD3^−^CD56^bright^ = 1.06%, CD3^+^CD16^+^ = 73.8 cells/mm^3^, NKT-cells = 50.3 cells/mm^3^, CD3^+^ = 4590.5 cells/mm^3^, TCRα*β* ^+^ = 3991.6 cells/mm^3^, TCR*γ* *δ* ^+^ = 452.4 cells/mm^3^, TCD4^+^ = 2850.2 cells/mm^3^, TCD8^+^ = 1862.6 cells/mm^3^, TCD4^+^CD8^+^ = 38.8 cells/mm^3^, TCD4^+^DR^+^ = 466.5 cells/mm^3^, TCD8^+^DR^+^ = 1471.6 cells/mm^3^, BCD19^+^ = 1523.0 cells/mm^3^, BCD5^+^ = 851.3 cells/mm^3^, BCD5^−^ = 576.7 cells/mm^3^, and BCD23^+^ = 959.0 cells/mm^3^. Once the cut-offs for each biomarker were established, we selected infants with high biomarker counts and assembled the data using gray-scale diagrams to calculate the frequency of those for each clinical group. Relevant data (>50%) were then highlighted in bold/underline format. Radar charts were constructed to characterize the overall frequency of infants with high levels of a given innate or adaptive immune cell population. GraphPad Prism 5.00 software (San Diego, USA) was used for graphical arts.

#### 2.3.3. Biomarker Network

Biomarker networks were assembled to assess the association between cell subpopulations (monocytes, NK-cells, NKT-cells, T-cells, and B-cells) and their subsets for each clinical group. The correlations were significant when Spearman's test resulted in a *P* < 0.05. Significant correlations representing the interaction between biomarkers tested were compiled using the open source software Cytoscape (version 2.8; http://www.cytoscape.org), as previously reported [12]. Biomarker networks were constructed using circle layouts with each biomarker being represented by a specific cartoon (monocytes; NK- and NKT-cells; T-cells and B-cells). Connecting edges display the underscore as negative, moderate, and strong, as proposed previously [13]. The correlation index (*r*) was used to categorize the correlation strength as negative (*r* < 0), moderate (0.36 > *r* < 0.67), or strong (*r* > 0.68). GraphPad Prism 5.00 software (San Diego, USA) was used for the data analysis.

## 3. Results

### 3.1. Leukocytosis with Increased Monocyte and Lymphocyte Counts is a Relevant Hematological Biomarker of Active Retinochoroidal Lesions in Congenital Toxoplasmosis

The analysis of hematological parameters demonstrated that TOXO is accompanied by relevant leukocytosis with increased monocyte and lymphocyte counts. Further categorization of infants, according to their ophthalmological records, showed that these changes were particularly observed in ARL patients. Relevant monocytosis was also observed in ACRL patients. No significant differences were observed in the NRL and CRL subgroups (Table 1).

### 3.2. Expansion of CD14^+^CD16^+^HLA-DR^high^ Proinflammatory Monocytes Is Present in Infants with Active Retinochoroidal Lesions in Congenital Toxoplasmosis

**Figure 1:**
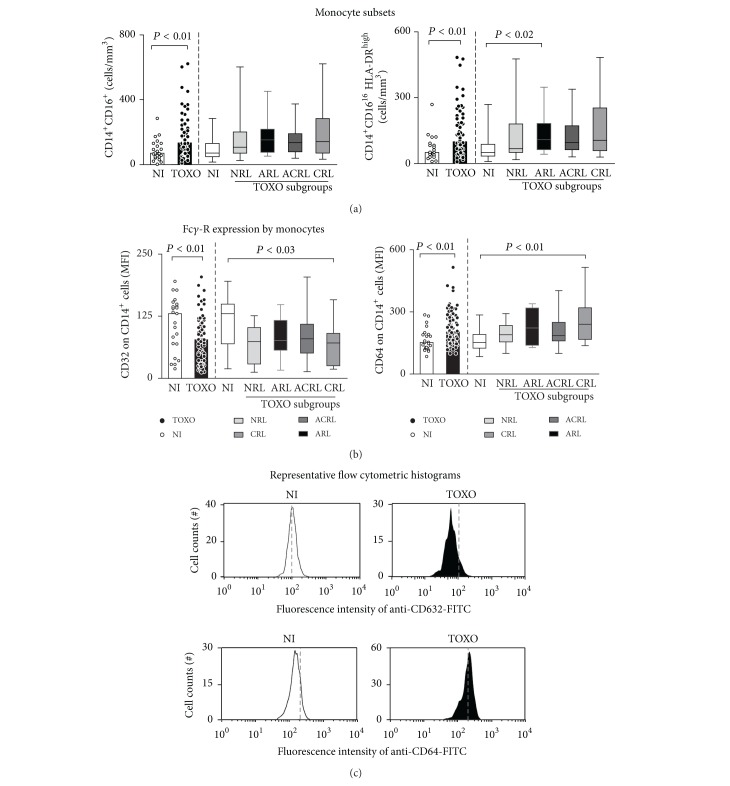
Monocyte subsets (a) and FC*γ*-R expression profile of monocytes (b) in the peripheral blood of congenital toxoplasmosis infants (TOXO) and noninfected controls (NI). Congenital toxoplasmosis was further categorized according to the clinical ocular status, referred to as no retinochoroidal lesions (NRL), active retinochoroidal lesions (ARL), active/cicatricial retinochoroidal lesions (ACRL), and cicatricial retinochoroidal lesions (CRL). The results are expressed as scatter plots of individual values and the medians of absolute cell counts/mm^3^ (left side) or in box-plot format (right side), where the box stretches from the lower hinge (25th percentile) to the upper hinge (75th percentile), and the middle half represents the median of the distribution as a line across the box. Significant differences are highlighted by connecting lines, and *P* values are shown in the figure. (c) Representative histograms illustrating the downregulation of CD32 (top panels) and the upregulation of CD64 (bottom panels) on circulating monocytes from infants with congenital toxoplasmosis (black) compared to noninfected controls (white). The dashed lines were set to highlight the shift in the histogram distribution toward lower or higher FC*γ*-R expression by monocytes.

When evaluating monocyte subsets, our results demonstrated that infants in the TOXO group presented an increase of CD14^+^CD16^+^ macrophage-like and CD14^+^CD16^+^HLA-DR^high^ proinflammatory monocytes (Figure 1(a)). In fact, the increase in CD14^+^CD16^+^HLA- DR^high^ proinflammatory monocytes was particularly observed in the ARL subgroup when compared with NI controls (Figure 1(a)).

Furthermore, analysis of FC*γ*-R expression by monocytes (Figure 1(b)) showed that TOXO displayed relevant changes in CD32 and CD64 expression on the surface of these cells. Analysis of the TOXO subgroups demonstrated that the CRL infants, in particular, had decreased CD32 and increased CD64 expression when compared with NI control group (Figure 1(b)).  Figure 1(c) shows representative flow cytometric histogram analyses of the CD32 and CD64 expression observed in the TOXO and NI groups.

### 3.3. NK- and NKT-Cell Subsets Are Expanded in the Peripheral Blood of Infants with Congenital Toxoplasmosis

**Figure 2:**
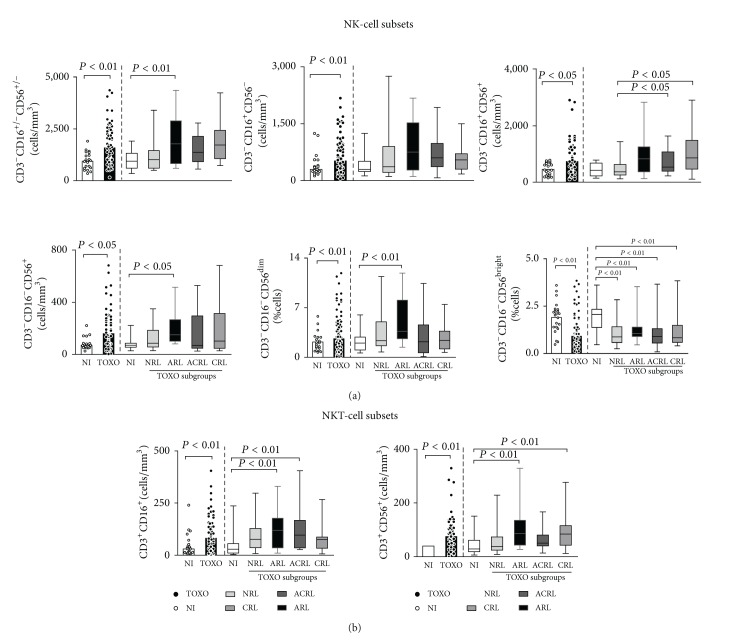
NK- (a) and NKT-cell subsets (b) in the peripheral blood of congenital toxoplasmosis infants (TOXO) and noninfected controls (NI). Congenital toxoplasmosis was further categorized according to the clinical ocular status, referred to as no retinochoroidal lesions (NRL), active retinochoroidal lesions (ARL), active/cicatricial retinochoroidal lesions (ACRL), and cicatricial retinochoroidal lesions (CRL). The results are expressed as scatter plots of individual values and the medians of absolute cell counts/mm^3^ (left side) or in box-plot format (right side), where the box stretches from the lower hinge (25th percentile) to the upper hinge (75th percentile), and the middle half represents the median of the distribution as a line across the box. Significant differences are highlighted by connecting lines, and *P* values are shown in the figure.

Data regarding total CD3^−^CD16^+/−^CD56^+/−^ NK-cells revealed an increased absolute count in peripheral blood samples from the TOXO group. Analysis of NK-cell subsets showed an increase in all NK-cell subsets in the TOXO group, including CD3^−^CD16^+^CD56^−^, CD3^−^CD16^+^CD56^+^, and CD3^−^CD16^−^CD56^+^ cells (Figure 2(a)). Analysis of TOXO subgroups demonstrated that whereas total CD3^−^CD16^+/−^CD56^+/−^ NK-cells were enhanced in the ARL subgroup, CD3^−^CD16^−^CD56^+^ NK-cells were expanded in the ACRL and CRL subgroups compared with the NI and NRL patients, respectively (Figure 2(a)).

Analysis of NKT-cell subsets showed a significant increase in both the CD3^+^CD16^+^ and CD3^+^CD56^+^ cell subpopulations in the TOXO group compared with the NI group. In fact, CD3^+^CD16^+^ cells were particularly expanded in the ARL and ACRL subgroups, whereas CD3^+^CD56^+^ cells were significantly increased in the ARL and CRL subgroups compared to NI controls (Figure 2(b)).

To characterize the major phenotypes related to the distinct functional features of NK-cells, we investigated the frequency of CD56^dim⁡^ cytotoxic and CD56^bright^ immunoregulatory cell subsets. Our findings demonstrated a decreased percentage of CD3^−^CD16^+^CD56^bright^ cells along with the expansion of CD3^−^CD16^+^CD56^dim⁡^ cells in the TOXO group compared to the NI group. Additional analysis revealed that CD3^−^CD16^+^CD56^bright^ cells were reduced in all TOXO subgroups, while CD3^−^CD16^+^CD56^dim⁡^ cells were particularly expanded in the ARL subgroup compared to the NI group (Figure 2(a)).

### 3.4. Augmented Activation of CD4^+^ T-Cells is Closely Related to the Presence of Active Retinochoroidal Lesions

**Figure 3:**
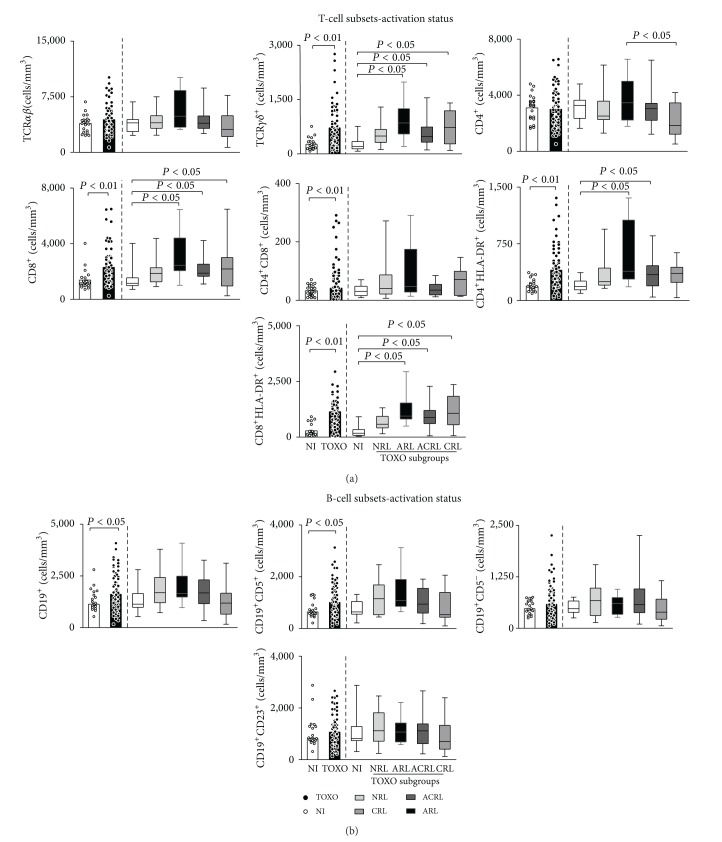
Major subsets and activation status of T- (a) and B-cells (b) in the peripheral blood of infants with congenital toxoplasmosis (TOXO) and noninfected controls (NI). Congenital toxoplasmosis was further categorized according to the clinical ocular status, referred to as no retinochoroidal lesions (NRL), active retinochoroidal lesions (ARL), active/cicatricial retinochoroidal lesions (ACRL), and cicatricial retinochoroidal lesions (CRL). The results are expressed as scatter plots of individual values and the medians of absolute cell counts/mm^3^ (left side) or in box-plot format (right side), where the box stretches from the lower hinge (25th percentile) to the upper hinge (75th percentile), and the middle half represents the median of the distribution as a line across the box. Significant differences are highlighted by connecting lines, and *P* values are shown in the figure.

Analysis of the adaptive immunity compartment is presented in Figure 3. Our data showed that TOXO patients presented increased counts of activated CD4^+^HLA-DR^+^ T-cells along with CD4^+^CD8^+^ T-cells, CD19^+^ B-cells, and CD19^+^CD5^+^ B1-cells compared to NI patients (Figures 3(a) and 3(b)).

Additionally, our data demonstrated that CD4^+^ T-cell counts were increased in the ARL subgroup compared to the CRL subgroup. Moreover, CD4^+^HLA-DR^+^ cells were particularly increased in the ARL and ACRL subgroups compared to the NI group (Figure 3(a)). No changes in B-cell subsets were observed among the TOXO subgroups (Figure 3(b)).

Our data demonstrated increased counts of TCR*γ* *δ* ^+^ cells, CD8^+^ T-cells, and particularly activated CD8^+^HLA-DR^+^ T-cells in the TOXO group. Analysis of TOXO subgroups demonstrated that TCR*γ* *δ* ^+^ cells, CD8^+^ T-cells, and activated CD8^+^HLA-DR^+^ T-cells, in particular, were increased in all groups with retinochoroidal lesions (ARL, ACRL, and CRL).

### 3.5. Expanded Frequency of Infants with High Levels of Innate and Adaptive Immune Cells Characterizes the Biomarker Signature Associated with the Presence of Active Retinochoroidal Lesions

**Figure 4:**
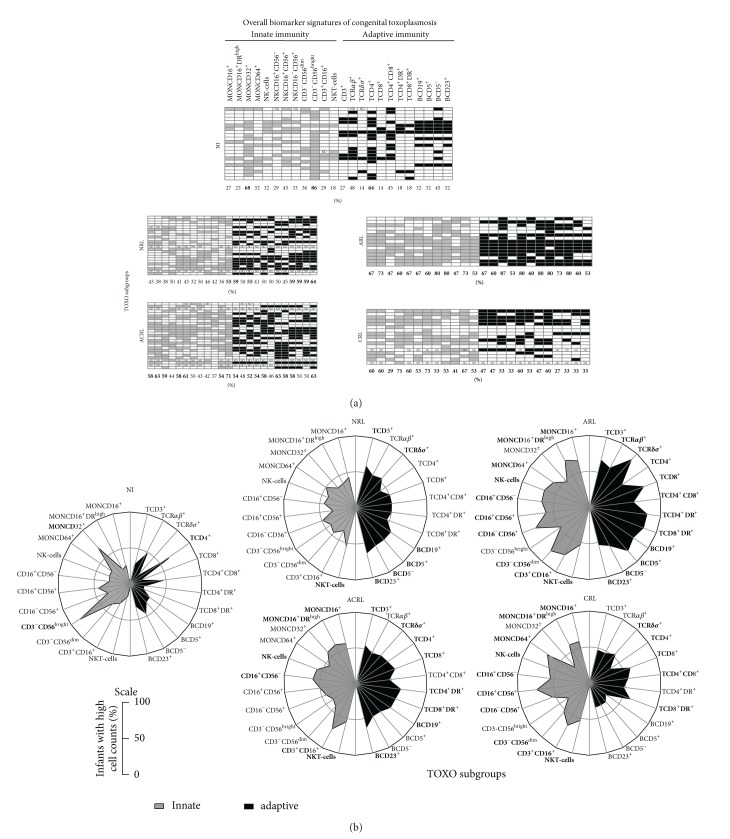
Biomarker signatures of innate and adaptive immunity in the peripheral blood of infants with congenital toxoplasmosis categorized as no retinochoroidal lesions (NRL), active retinochoroidal lesions (ARL), active/cicatricial retinochoroidal lesions (ACRL), and cicatricial retinochoroidal lesions (CRL) compared to noninfected controls (NI). (a) Gray-scale diagrams were assembled using the global median value of each cell subset as the cut-off mark to tag each infant as presenting “low” or “high” (innate; adaptive) levels of a given cell population. The frequency (%) of infants displaying high cell counts is provided, and the relevant data (>50%) are underscored in bold/underline format. (b) The radar charts summarize the biomarker signature of innate and adaptive immunity, where each axis displays the proportion of infants with high levels of a given cell population. The relevant data (>50%) are underscored in bold/underline format.

To further characterize the immunological profile associated with distinct clinical manifestations of congenital toxoplasmosis, we assembled the overall phenotypic biomarker signature of peripheral blood innate/adaptive immune cells using the innovative/nonconventional data analysis approach referred to as the biomarker signature of innate and adaptive immunity compartments in the peripheral blood of infants with congenital toxoplasmosis (Figure 4).

Our data demonstrated that, in the NI group, most biomarkers were confined to frequencies below 50%, except MONCD32^+^, CD3-CD56^bright^, and CD4^+^ T-cells. However, in all TOXO subgroups, most biomarkers were confined to frequencies above 50%. Specifically, the NRL subgroup predominantly showed enhancement of B-cell related biomarkers (BCD19^+^, BCD5^+^, BCD5^−^, and BCD23^+^) along with TCD3^+^, TCR*γ* *δ* ^+^, and NKT-cells in the innate immune compartment. By contrast, the CRL subgroups predominantly showed increases in the frequency of innate immunity biomarkers (MONCD16^+^, MONCD16^+^DR^high^, MONCD64^+^, NK-cells, CD16^+^CD56^−^, CD16^+^CD56^+^, CD16^−^CD56^+^, CD3^−^CD56^dim⁡^, CD3^+^CD16^+^, and NKT-cells but not MONCD32^+^ and CD3^−^CD56^bright^) along with T-cell related biomarkers (TCR*γ* *δ* ^+^, TCD4^+^, TCD8^+^, TCD4^+^CD8^+^, and TCD8^+^DR^+^).

The profile of the ARL subgroup was of particular interest, with an enhanced frequency of biomarkers in both the innate and adaptive immune compartments. All biomarkers included in this investigation were found to be increased to above 50% in the ARL infants evaluated, except for MONCD32^+^ and CD3^−^CD56^bright^. The ACRL subgroup showed an overall biomarker signature similar to that observed in the ARL subgroup, with minor changes mainly in the NK- and B-cell subsets.

### 3.6. Remarkable Lack of Connections Involving B-Cells Is Observed in Infants with Active Retinochoroidal Lesions

**Figure 5:**
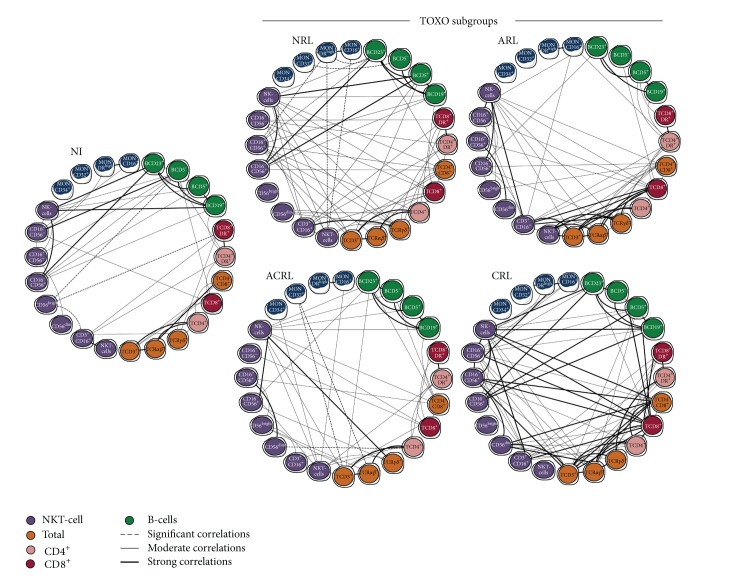
Biomarker networks of innate and adaptive immunity in the peripheral blood of infants with congenital toxoplasmosis categorized as no retinochoroidal lesions (NRL), active retinochoroidal lesions (ARL), active/cicatricial retinochoroidal lesions (ACRL), and cicatricial retinochoroidal lesions (CRL) compared to noninfected controls (NI). Networks were assembled to assess the association between leukocyte subpopulations, including monocytes, NK- and NKT-cells, T-cells (total, CD4^+^, and CD8^+^), and B-cells. Significant correlations at *P* < 0.05 are highlighted by connecting edges to underscore negative, moderate, and strong indexes.

Exploratory analysis of biomarker networks demonstrated that although some axes intrinsic of innate and adaptive immunity were preserved in all clinical groups, some connections were lost in the TOXO subgroups (Figure 5).

In the NI group, NK- and B-cells clearly represent relevant foci of connections. A relevant shift of NK-cell connections toward T-cells was observed in the ARL subgroup along with a selective loss of connections with the B-cell compartment (Figure 5).

The ACRL subgroup clearly showed a transitional profile between the ARL and CRL subgroups with NK-cell connections focusing on T-cells and restoring the connections with B-cells (Figure 5).

### 3.7. Complex and Imbricated Biomarker Networks Underscore the Interaction of Monocytes with NK- and B-Cells in Protective Events, Whereas NK-Cells and CD8^+^ T-Cells Appear Relevant to Mechanisms of Resolution

The fact that the NRL and CRL subgroups displayed a higher number of significant interactions and a more complex and imbricated biomarker network was outstanding. In fact, a rich number of connections were observed in these subgroups.

It was clear that the NRL subgroup showed relevant interaction between monocytes and other cell subsets (NKT-, NK-, and B-cells) mediated by negative correlations and a relevant role of NK-cell connections focusing on T- and B-cells (Figure 5).

In the CRL subgroup, a strong correlation axis could be identified, with the pivotal participation of several NK-cell subsets interacting with T- and B-cells along with strong connections of CD8^+^ T-cells with a broad range of cell subsets (Figure 5).

## 4. Discussion

Retinochoroiditis in humans caused by* T. gondii* is the most frequent clinical manifestation of congenital and acquired parasite infection [14, 15]. The disease typically presents as a unilateral focal necrotizing lesion in the presence of adjacent scars [1].

The common occurrence of toxoplasmic retinochoroiditis is believed to be under the influence of the status of the host immune response [16], the genotype of infective parasite strains [2], and the host genetic background [17, 18]; however, the participation of cellular components that lead to the establishment of this ocular manifestation has not been addressed well in humans.

The presence of active, active/cicatricial, or cicatricial lesions observed in the infants of our cohort is consequence of multiple factors, which may include parasite virulence and retinotropism of* T. gondii* in Brazil, individual susceptibility as well as the* T. gondii*-specific immune response in the infants of our cohort. Another possibility is that the time of infection during pregnancy may impact the outcome of distinct retinochoroidal lesions. The premise that putative infection by parasites with diverse virulence and/or atypical/recombinant genotypes leads to different manifestation of ocular toxoplasmosis was not confirmed by our parasitology team, as shown by Carneiro et al., 2013 [19]. Moreover, preliminary results regarding IgM reactivity as well as IgG avidity do not support the hypothesis that time of infection during pregnancy may influence the occurrence of distinct patterns of retinochoroidal lesions (paper in preparation). The development of an adequate innate immune response is important for infection control and reduction of toxoplasmosis-associated injuries, primarily retinochoroiditis. However, the initial stages of congenital infection are unclear, and most studies mainly refer to work with experimental models [20–22]. The present study is a great opportunity to understand the major and minor changes in peripheral blood leukocyte subpopulations in infants with congenital toxoplasmosis. Although the analysis of* in vitro T. gondii*-specific immune response is highly relevant to understand the role of the parasite-derived antigens essential to elucidate the mechanism underlying the immunopathogenesis of ocular toxoplasmosis, we believe that the* ex vivo* analysis particularly in the absence of exogenous stimuli is highly relevant to map the events that take place* in vivo* and may point out putative biomarkers useful to comprehend the systemic network involved in the immunopathogenesis of ocular toxoplasmosis.

Infants were selected through a newborn screening program, and therefore, no information about gestational age when the congenital infection occurred was available. Our results showed an increase in the populations of monocytes and lymphocytes in infants with congenital toxoplasmosis. It is known that monocyte recruitment is essential in restricting the growth of* T. gondii* in murine models of toxoplasmosis [6, 23]. However, other studies show that these cells as well as dendritic cells are strong candidates for the intracellular transport of* T. gondii* in the blood as a “Trojan Horse” [24]. Moreover, the activity of monocytes must be carefully controlled because excessive production of inflammatory cytokines and nitric oxide (NO) can result in severe immunopathology [25]. The increase in the population of monocytes was observed only in the active lesion groups, suggesting that the persistence of the immune response mediated by monocytes is directly related to the pathology observed in these infants.

After this previous analysis, we examined the changes in specific monocyte subsets. An increase in proinflammatory monocytes (CD14^+^CD16^+^HLA-DR^++^) in infants with congenital toxoplasmosis was observed. Previous studies have shown that inflammatory monocytes produce IL-12* in vitro* and* in vivo* when stimulated with* T. gondii* [6, 7], and it has also been proposed that these cells contribute to the direct control of* T. gondii* through the production of NO, which inhibits parasite replication [8]. Increased proinflammatory monocytes in infected infants, particularly in those with active retinochoroidal lesions, are indicative of a strong and persistent proinflammatory response.

NK-and NKT-cells are other innate populations involved in immunity against* T. gondii* that are increased in infants with congenital toxoplasmosis compared with noninfected children. Our results showed an increase in the population of CD56^dim⁡^ cells in infants with active lesions when compared with an important decrease in the immunoregulatory NK subset, expressing CD56^bright^. The increase in this population and in subsets of NK- and NKT-cells is an important feature of the innate immune responses against the parasite [26].

Analysis of the adaptive immune response showed that CD4^+^ T lymphocytes are apparently associated with the active lesion phenotype. Although several studies have demonstrated the importance of CD4^+^ T-cells in infection control, the production of proinflammatory cytokines, mainly by CD4^+^ T cells, is related to the morbidity of toxoplasmosis [27, 28]. Moreover, we observed that an increase in CD8^+^ T-cells was associated with the presence of retinochoroiditis in infants. CD8^+^ T-cells play a major role as effector lymphocytes against the parasite [29] and in killing infected macrophages and macrophages exposed to soluble parasite antigens [30]. Our results show that the increase in CD8^+^ T-cells could also be an important biomarker of morbidity in infected infants.

The results observed in infected infants suggest maturation of the immune response against* T. gondii*. The increases in lymphocyte populations and in subpopulations of monocytes and NK-cells are important in controlling parasitemia. However, exacerbation of the proinflammatory response may also be damaging to infants and, therefore, a determining factor in the pathology observed. Only infants with active lesions showed increased leukocyte counts (specifically monocytes, NK-cells, and lymphocytes). Moreover, the same children also showed increased subpopulations of proinflammatory monocytes and active NK-cells. It is important to consider, however, that future studies with proper validation of the immunological subsets are still needed in order to support their predictive value and specificity as putative biomarkers of ocular involvement in congenital toxoplasmosis.

## 5. Conclusion

Studies on the immune response in human infections by* T. gondii*, particularly in newborns, are rare. This work provides important findings regarding the immune response to congenital toxoplasmosis, which indicated immunomodulation possibly associated with local control of retinochoroiditis. Future studies with proper validation of the immunological subsets are still needed in order to support their predictive value and specificity as putative biomarkers in the ocular congenital toxoplasmosis.

## Figures and Tables

**Table 1:** Hematological records in infants with congenital toxoplasmosis∗.

Parameters	NI (*n* = 22)	TOXO (*n* = 76)	TOXO subgroups			
			NRL (*n* = 22)	ARL (*n* = 15)	ACRL (*n* = 24)	CRL (*n* = 15)
WBC	9.3 (5.3–17.3) × 10^3^	**11.6** (5.6–22.9) × 10^3^	11.0 (6.2–17.3) × 10^3^	**14.5** (7.6–23.0) × 10^3^	11.8 (5.7–19.4) × 10^3^	11.2 (5.7–18.2) × 10^3^
Monocytes	0.8 (0.1–1.9) × 10^3^	**1.2** (0.1–2.3) × 10^3^	1.1 (0.2–2.1) × 10^3^	**1.4** (0.5–1.7) × 10^3^	**1.3** (0.2–2.3) × 10^3^	1.0 (0.1–2.4) × 10^3^
Neutrophils	1.8 (0.7–4.0) × 10^3^	1.7 (0.5–5.7) × 10^3^	1.8 (1.0–3.0) × 10^3^	1.7 (0.8–3.6) × 10^3^	1.6 (0.4–5.3) × 10^3^	1.6 (0.9–5.7) × 10^3^
Lymphocytes	6.2 (3.9–14.2) × 10^3^	**7.5** (1.0–18.8) × 10^3^	7.3 (4.3–14.2) × 10^3^	**8.5** (5.4–18.1) × 10^3^	7.5 (3.9–13.9) × 10^3^	7.4 (1.0–13.9) × 10^3^
Eosinophils	0.4 (0.1–0.8) × 10^3^	0.4 (0.1–1.7) × 10^3^	0.4 (0.08–0.8) × 10^3^	0.5 (0.2–1.1) × 10^3^	0.4 (0.003–1.7) × 10^3^	0.3 (0.06–1.1) × 10^3^
RBC	3.5 (2.8–4.5) × 10^6^	3.6 (2.9–5.1) × 10^6^	3.5 (2.5–4.4) × 10^6^	3.68 (3.1–4.1) × 10^6^	3.5 (2.4–4.5) × 10^6^	3.71 (2.5–4.2) × 10^6^
Hb (g/dL)	10.8 (8.5–13.1)	10.5 (8.1–13.9)	10.3 (7.8–13.0)	10.8 (8.3–12.2)	9.9 (7.7–12.9)	10.75 (7.2–12.9)
Hct (%)	31.0 (25.4–39.4)	31.9 (22.1–45.4)	30.6 (23.0–39.1)	32.8 (27.1–37.3)	29.4 (20.8–37.7)	31.8 (23.0–36.9)
PLT	4.8 (1.3–7.3) × 10^5^	3.8 (1.1–7.9) × 10^5^	4.31 (1.4–6.7) × 10^5^	3.35 (1.4–6.3) × 10^5^	3.61 (1.4–6.3) × 10^5^	2.87 (1.7–4.9) × 10^5^

*WBC = white blood cells; RBC = red blood cells; PLT = platelets. Results are expressed in cell counts/mm^3^. Significant differences at *P* < 0.05 are highlighted by bold format.
